# Providing equity of care for patients with intellectual and developmental disabilities in Western Switzerland: a descriptive intervention in a University Hospital

**DOI:** 10.1186/s12939-019-0948-8

**Published:** 2019-03-18

**Authors:** Séverine Lalive d’Epinay Raemy, Adeline Paignon

**Affiliations:** University of Applied Sciences and Arts of Western Switzerland, School of Health Sciences, Geneva, Switzerland

**Keywords:** Intellectual disability, Developmental disability, Acute care setting, Hospital

## Abstract

**Background:**

The purpose of this article is to describe an interventional project in a University Hospital. It explains the adjustments that were made to provide good care for patients with intellectual and developmental disabilities in an acute care setting in Western Switzerland. It is not the exposition of the results of a formalised research or study. Rather, this article relates the success story of a project initiated by a small group of passionate people on their free time, that eventually entered in the 2020 strategic planning of the largest hospital of Switzerland.

Switzerland does not have a national policy regarding health needs for patients with intellectual and developmental disabilities. Health care professionals are not trained to identify and meet the specific health needs of this population and little is taught about intellectual and developmental disabilities during undergraduate studies.

**Method:**

The Disability Project was conducted between 2012 and 2017 in Geneva University Hospital, as follows:

Firstly, over sixty working group sessions took place to identify the specific health needs of people with intellectual and developmental disabilities, to identify the barriers to providing equity of care and to prioritize reasonable adjustments. The four following barriers emerged from these meetings:

1. Lack of awareness of healthcare professionals on specific health issues for patients with intellectual and developmental disabilities, which resulted in a poor healthcare coordination and reduced quality of care.
2. Communication and information transmission issues between hospital staff, families and supported residential accommodations.
3. Lack of training or insufficient training of healthcare professionals and hospital staff on intellectual and developmental disabilities.
4. Inaccessibility of the hospital facilities and buildings for patients with disabilities

Secondly, arising from these priorities, interventions were developed.

**Findings:**

The interventions were eventually applied throughout the hospital. Recommendations and reasonable adjustments were made to provide accessibility and equity of care for patients with intellectual and developmental disabilities.

**Conclusion:**

The Disability Project has achieved many reasonable adjustments in an acute care setting to provide good care and satisfaction for this population and their families.

## Background

One in seven people experience disability in the world [1]. The World Health Organisation (WHO) states that “people with disabilities have generally poorer health, lower education achievements, fewer economic opportunities and higher rates of poverty than people without disabilities” [1]. A disabled person is twice as likely to find healthcare providers’ skills and facilities inadequate, three times more likely to be denied healthcare and four times more likely to be treated badly in the healthcare system [2]. This statement is corroborated by the Mencap [3, 4] and Michael-Richardson reports [5] in the United Kingdom, a country which shares with Switzerland, Canada and France a similar issue of access to healthcare inequalities for PWID. These rather concerning statements should be an incentive to health providers to raise awareness among HealthCare Professionals (HCP) in Switzerland. Wieland [6] indicates, regarding the hospitalisation of patients with intellectual and developmental disabilities (PWID) in Swiss Hospitals, that between 1997 and 2008 only 0.2% of the PWID were hospitalised every year, while 1% of the general population was hospitalised per year. Statistically, they should have the same ratio of hospitalisation as the general population. This shows that 0.8% of the PWID have either not been identified as being disabled or worse, might not have the same access to healthcare as the general population in Switzerland. Furthermore, the Federal Office of Statistics [7] states that, PWID (between the age of one to forty-four years), who are institutionalised, have a six to nine times higher rate of mortality than the general population. This due to their disabilities and poorer health conditions.

According to the WHO Health Report [8] and Maulik [9], the overall prevalence of intellectual and developmental disability (ID) is between 1 and 3%. The Swiss population being 8,4 million people, it is estimated that 80,000 to 240,000 people are in this population and hence concerned by accessing healthcare. It can be extrapolated that Geneva, having 490,000 inhabitants, has approximately 12,800 people living with an ID. The life expectancy of PWID has increased notably to practically match the life expectancy of the general population, hence they are exposed to the same health issues as every ageing person, with greater risks to develop diseases and new disabilities [10, 11]. The cost of healthcare for PWID cannot be precisely estimated in Switzerland due to the lack of accurate data concerning health care. But with the cost of health for the general population being CHF 77.754 million in 2015 [12], it can be estimated that 1 to 3% of this sum applies to the health care of PWID, that is CHF 777 to 2332 million. 34,9% of the total sum that is spent on hospitals (CHF 27,158 million). This demonstrates that the subject is of considerable importance.

Apart from the financial perspective, the legal aspects justify such a project as well. Indeed, the Convention on the Rights of Persons with Disabilities [13] requires that parties should provide the same range, quality and standard of care for PWID as for the general population. It is common belief, in Switzerland, that they have the same access to mainstream health care services as the general population. However, families, associations, social care providers and representatives of supported residential accommodations make the same assessment as the WHO: PWID do not get the same quality of healthcare as the general population, they have less access to healthcare services and therefore their healthcare needs are not met [6].

The training of the HCP participates greatly in the quality of care that PWID receive. In fact, providing good care to this specific population requires specific skills and knowledge, particularly in communication, legal rights, pain detection, and identification of atypical clinical symptoms [14, 15]. Providing good care for a person with challenging behaviour in an emergency service can be very difficult if the HCP has not been trained for it [16, 17]. The literature shows evidence that the training of the HCP on ID is a crucial factor in providing good care for PWID [2, 15, 18].

Generally, the literature makes the following recommendations to achieve equity and quality of care for PWID: Both the reports from the “Union Nationale des Associations de Parents, de personnes handicapées mentales et de leurs amis” (UNAPEI) [18] and from the “Académie Suisse des Sciences Médicales” (ASSM) [14] recommend that PWID should be included in prevention and health promotion programs; that specialized and referral centres and professionals should be developed; that a specialised physician and nurse should be identified in acute care settings as referral and support; and that a coordinated care path should be organised [14, 18–20]. Other recommendations are that HCP should be trained on ID and legal rights [1]; that social workers, care workers should be trained on health issues for PWID [21, 22]; that family care givers also should be trained on health issues; that communication between all partners be improved through documents, such as health passports and documents that are easy to read and understand [23]; and finally, that epidemiologic studies on PWID’s specific health needs should be promoted [24, 25]. There is an important knowledge gap on the subject in Switzerland and other European countries due to lack of awareness on this issue hence the non-existence of specific data. So far, the hospital admission registration has not tracked the degree of intellectual disability of the patient, if any. This means it is impossible to relate the history of interventions to the population of PWID.

The aim of the Disability Project is to provide good care for PWID in an acute care setting. It is a challenge that the project team has decided to address to reach equity and quality of care for PWID in Switzerland.

## Method

### Location

The Disability Project was conducted between 2012 and 2017 in a University Hospital of approximately fifteen hundred beds, the “Hopitaux Universitaires de Genève” (HUG). It was decided to start the project with the emergency service (ES) (before further extension) because, most of the time, the ES is the entry point of the hospital for PWID. It is the right place to identify them and, as such, the ES was the environment of choice to test and implement the “reasonable adjustments”.

Later on, the project was extended, first to the intern medicine department, and then to other specific units where PWID were more likely to be hospitalised, such as neurology and intensive care services.

### Population

The targeted patient group of the project, patients with intellectual and developmental disabilities, autistic spectrum disorders and severe disabilities, (PWID) was chosen because of its extreme vulnerability and unmet health needs as stated by the WHO [26]. One of the main difficulties encountered by the project leader was the lack of data regarding PWID in the hospital. There were no statistics on how many PWID were hospitalised or for how long. As there was no accurate medical coding system or other way to identify this group, it was decided to identify PWID according to their supported residential accommodations or institutions, as well as via associations of parents of PWID. No other criteria were added for fear of raising a feeling of segregation or exclusion.

### Team

This project gathered professionals from many fields of expertise and made them work together towards the same purpose. The multidisciplinary project team consisted of nurses, medical doctors, physiotherapists, senior nurses, social workers, representatives of families, associations, architects, representatives of the main supported residential accommodations and lecturers from the Nursing department of the University of Applied Sciences, all volunteers, motivated by the desire to provide good care for PWID.

### Process

This Disability Project was organised in three phases:

#### Pilot phase

A kick-off was organised by the Nurse Project Manager and the Medical Chief of the Intern Medicine department, the Head Nurse of the Emergency Service and a Senior Case Manager Nurse, they constituted the leadership team. They invited every known partner in the city to a multidisciplinary meeting for a brainstorming: it was open to families, residential care professionals, doctors, nurses, hospital staff, social workers and parents’ associations. Every possible partner was invited to be included in the project.

The leadership team decided to meet the barriers identified from the multidisciplinary meeting, by establishing four working groups (with hospital staff and family’s representatives), each with a specific purpose. They inventoried the existing competencies, identified interdisciplinary professionals and staff members who could add value to the project. Significant reports such as: Mencap, Michael-Richardson, UNAPEI, CRPD, WHO, HAS and Bowness have identified the barriers in a wider international context. The leadership team compared those barriers and reasonable adjustments to those experienced by families and professionals.

The following difficulties were gathered during the first multidisciplinary meeting: Parents and care workers complained that there were not listened to when arriving at the emergency service; the specific needs of their adult child were often not considered; they were not treated adequately, sometimes they were denied healthcare when their adult child, with severe autism or challenging behaviour, would not comply to an examination. Too often they were sent home or to their supported residential accommodation without proper examination, without information about what had been done or which treatment had been given. They were sometimes sent home in the middle of the night with no information, anticipation nor continuity of care. During hospitalisation, families and care workers were too often used as health carers and given huge responsibilities, such as removing the stitches of a patient with ID who would not agree to be touched by hospital staff. They were asked to wash and feed the patient with ID. They would have to repeat over again the same information. The challenging behaviours would be interpreted as a refusal of care, as a psychiatric condition or only related to the disability, instead of being taken as the symptom of a real somatic disease. Pain was often not considered, for the HCP did not have a pain scale adequate for a nonverbal patient. Environmental barriers were also mentioned: for instance, the lack of wider parking spots suitable for large wheelchair-enabled vehicles, or the revolving doors at the main entrance which were not usable with large electrical wheelchairs.

On the other hand, HCP complained that they were not trained to care for people with ID, autism or challenging behaviours, that they had not enough time (particularly in emergency services) to read the large medical files of the patient and to find the information they needed rapidly. They also confessed often feeling helpless and even sometimes being scared by PWID.

#### Phase one

Then the most important issues identified by the multidisciplinary meeting could be prioritized.

Each of the four working group was organised to identify the reasonable adjustments that would meet the barriers and prioritise the interventions. They met once a month, the project manager participating in each meeting and being responsible for centralising the intervention proposals of the four working groups. In total, over sixty working group sessions of 6 to 14 participants took place for a duration of 2 hours.

#### Phase two

Out of these priorities, several interventions were determined, the “reasonable adjustments”, each of them focusing on a different theme and purpose. To define what these reasonable adjustments should be, “the following factors could be considered: the effectiveness of the proposed adjustment, the practicality of the step, the financial and other costs incurred, any disruption caused and the extent of the organisation’s financial and other resources” [27]. That is, the “reasonable adjustments” are those that maximize efficiency while requiring a minimal financial and organisational effort.

#### Annual steering of the interventions

At the start of the project it was impossible to know what the needs of PWID were when admitted in the Emergency Service or how many were admitted in the ES or even hospitalised. The first adjustments made were a simplified admission procedure to the ES, a shortened waiting time and a quiet and dedicated waiting area. We will discuss later other reasonable adjustments that have been implemented in the ES and throughout the hospital.

From 2012 to 2017, five multidisciplinary meetings took place with all the partners to share the problems encountered when PWID are hospitalised. They met for an annual review. The leadership team was requested to make a quarterly report to the director of the hospital.

**Fig. 1:**
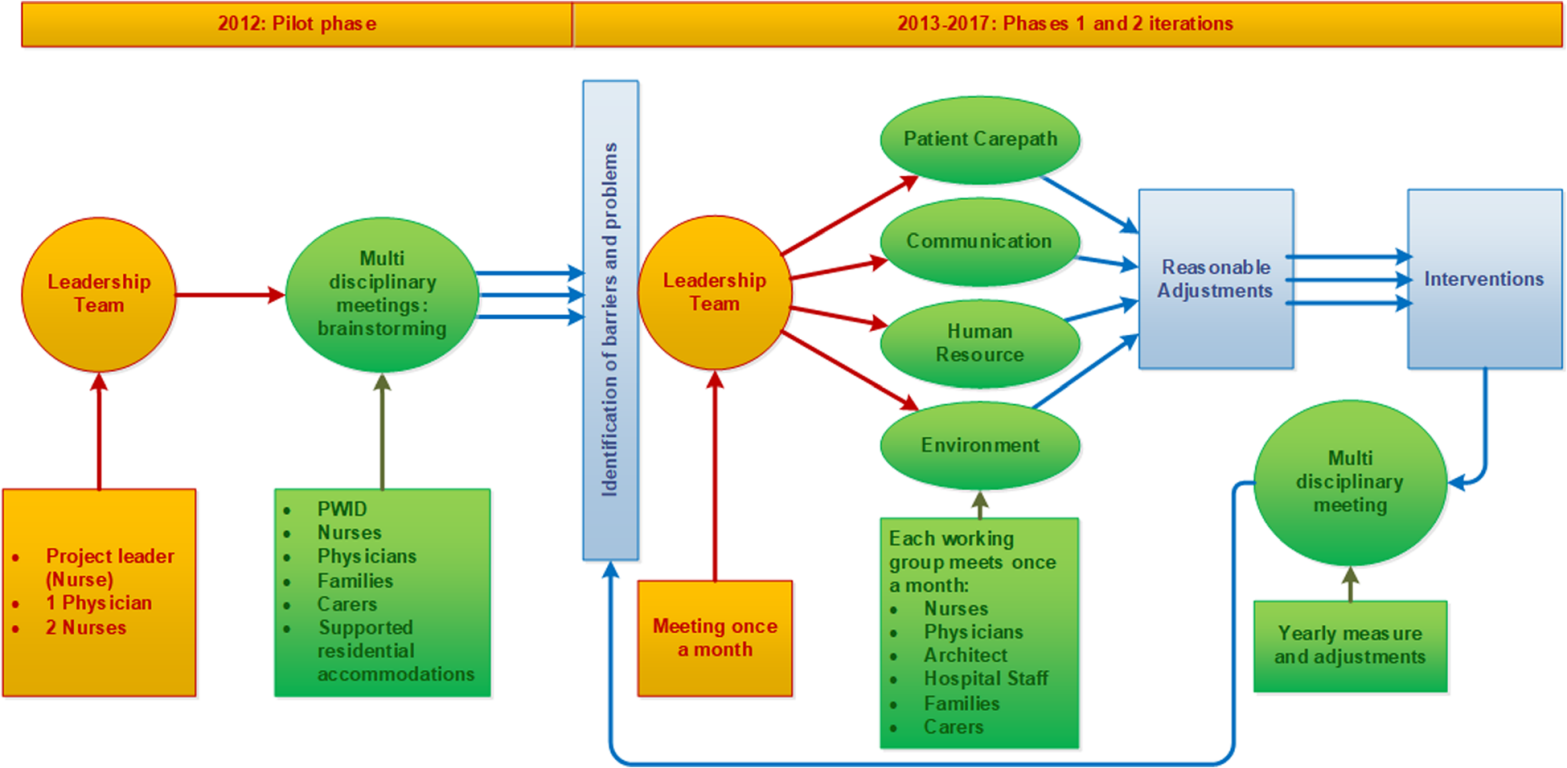
Pilot phase and yearly phases iterations. This figure describes the pilot phase, the phases one and two and the iteration of the process of the Disability Project

Every year each working group’s official report was documented and analysed according to the most recurrent barriers and the implementation of reasonable adjustments. The leadership team’s responsibility was to verify that the interventions were in line with the literature review and the expectations of the project partners Fig. 1.

## Interventions

### Barriers identification and determination of interventions

According to Sowney & Barr [16] “the barriers that reduce the opportunities for people with learning disabilities to have equal access to acute secondary healthcare services have been identified to include poor communication; a reduced quality of care; lack of carer’s confidence in nursing staff to provide care and nursing staff lacking in competence to carry out some clinical skills”. The UNAPEI report [18] identifies the barriers to equity of care as: 1.Poor communication; 2.Poor healthcare coordination; 3.Insufficient access to health prevention and reduced care; 4.Health professionals poorly trained; 5.Lack of accessibility. This is in line with the findings of our brainstorming meeting.

Hogg, et al. [28] state that “Access of persons with intellectual disability to primary health care provision may be restricted by a wide range of factors, among them: lack of training on intellectual disability itself, lack of training on health issues relative to older persons with intellectual disabilities, lack of pertinent information on the medical history of the individual, difficulties in undertaking medical examination because of communication problems or behaviour problems, absence of specialized back-up for complex medical conditions, and lack of understanding on the physicians part concerning informed consent issues.”

In this project, each of the working groups worked on a specific purpose, addressing one of several identified barriers:

1. Patient care path organisation and best practice: This working group was tasked to raise awareness on the inequity of healthcare and unmet specific health needs for PWID, as well as to find ways of improving the reception of PWID both physically and psychologically, mainly in the ES, but also in the different units of the hospital, including the out-patient clinics. Starting with a review of the existing best practice guidelines to promote consistency of care inside and outside the hospital, the purpose was to respond to the barriers identified by families and care workers such as inadequate care and insufficient pain medication

*Barriers: Reduced quality of care, poor Healthcare coordination.*

2. Communication: This working group was tasked to improve transmission of relevant information between the different partners inside and outside the hospital. Particularly, by formalizing an information transmission system common to PWID, families and professionals. Another responsibility of this group was to raise awareness amongst the hospital staff

*Barrier: Poor communication & awareness.*

3. Human resources: This working group was tasked to train the HCP in the most sensitive departments. This encompassed the creation of a network of professionals, expert in ID for consultation

*Barrier: Lack of training of healthcare professionals.*

4. Environment: This working group was tasked to inventory the existing resources and the equipment dedicated to PWID, to review and suggest access improvements to the facilities and buildings of the hospital

*Barrier: Inaccessibility.*

### Interventions

Figure 2

**Fig. 2:**
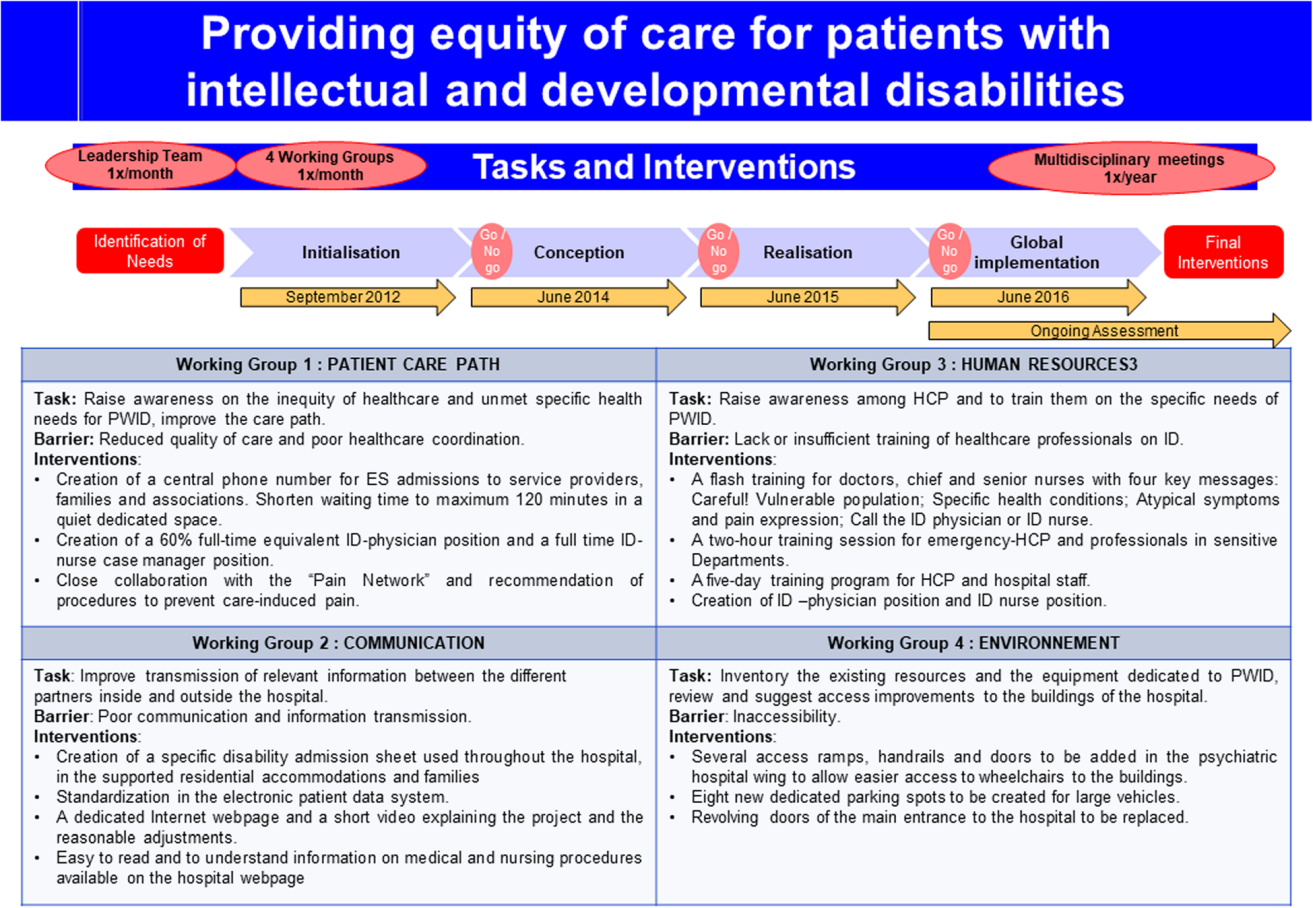
Summary of the interventions and reasonable adjustments achieved throughout the Disability Project

### Patient care path organisation

Providing good care for a person with challenging behaviour in an emergency service can be difficult, even sometimes impossible if HCP do not have the right information [16]. Particularly for PWID, it is essential to get the right information from the start, because they are more at risk of decompensation and then impossible to provide care for. In order to improve the communication between HCP, service providers and families, the following reasonable adjustments are made:

- A simplified admission procedure to the ES.
- A central phone number for ES admissions was opened and is available to service providers, families and associations.
- The waiting time in the ES has been reduced to a maximum of two hours, in a dedicated and quiet space.
- The use of a specific emergency admission sheet, developed by the Communication working group.
- In collaboration with the Human Resources working group, a 60% full-time equivalent ID-physician position was created for two years. Her role is to improve hospitalisation conditions, to coordinate the transition of paediatric to adult hospitalisation, resolve conflicts, support, guide and train HCP, collect data and lead research on ID.
- A close collaboration with the “Pain Network”, a specialized team of physicians and anaesthetists, was initiated from the start of the project. The pain relief procedures were modified and procedures such as administration of nitrous oxide and local anaesthetics to prevent care-induced pain are now recommended.

### Information and communication

Communication has been recognized as the key to providing good care for PWID in an acute setting. The purpose of this group was to find ways to facilitate the transmission of information between HCP and service providers. The specific emergency admission sheet was created. This document fits on a double-sided page and should be quick to read. It includes important specific information such as: type of disability, description of challenging behaviour, other impairments, legal representation, capacity of consent, how the person communicates and expresses pain, comfort, discomfort and anger. This admission sheet is standardized in the electronic patient data system, so that the relevant information is readily available to every HCP when needed.

This particular document relieves the parents and carers from repeating over again relevant information and helps the hospital staff quickly get the right information.

A more comprehensive document was developed, to collect further fundamental data regarding the patient in case of a hospitalisation of a PWID. It includes details such as specific care, preferences, dislikes, communication skills, specific health issues, usual behaviour and presence time organisation between the different partners, ensuring that roles and resources are clearly determined.

A dedicated internet webpage [29] was created on the public site of the hospital. It shows all the procedures, improvements, and provides with links to the ES admission sheet and “easy to read and understand” information on many medical procedures.

A short video explaining the project and the reasonable adjustments was produced, which makes PWID’s health needs and rights visible.

### Human resources

This working group had two main missions that were addressed simultaneously: to raise awareness among HCP and to train them on the specific needs of PWID.

To raise awareness on specific health issues for PWID, an innovative training program was developed and implemented through three levels of awareness with the participation of a disabled person as co-trainer and expert, as well as the use of a disability simulator. The training program includes:

- A flash training of fifteen minutes for doctors, chief and senior nurses with four key messages: Careful! Vulnerable population; Specific health conditions; Atypical symptoms and pain expression; Call the ID physician or nurse if needed!
- A two-hour session for emergency-HCP and professionals in sensitive departments (neurology and intensive care services).
- A five-day training program for HCP and hospital staff. The contents include intellectual and developmental disability, autistic spectrum disorders, rights and consent, self-determination, communication and pain identification, challenging behaviours, specific health issues and dental care.

On a global scale, a specific training program for student nurses was developed alongside in the University of Applied Sciences and Arts of Western Switzerland. It became obvious that it was important to train future HCP on the specific health issues of PWID during their primary training period.

To overcome the different barriers identified, a leader role was necessary, hence the creation of an ID-physician position. This new position was set for two years, with the support of a private funding. A specialized nurse position was also created a year later. The ID physician and ID-nurse now play a key role in:

- Adapting the various medical and care procedures to PWID.
- Coordinating the interventions, specific examinations and surgeries.
- Training healthcare providers and hospital staff members.
- Facilitating and improving communication between PWID, families, HCP and care providers.
- Collecting data, reviewing diagnostic codes and conducting researches on ID’s health issues in the future.

These various measures and adjustments contribute to improving the training of HCP, educating future HCP to address the specific health needs for PWID and advocating for equity of care for all.

### Environment

The architectural design of the HUG dates from the 1960s. The project helped to identify and prioritize the interventions according to three criteria: security, accessibility and comfort. First, an assessment was made on the accessibility of all buildings of the HUG and on the equipment for disabled people. Second, architectural changes were suggested and decided on a large scale to allow a long-term vision of the architectural strategic plan of the HUG. A member of this working group has joined the architectural committee on decisions regarding the architectural evolution of the future hospital and its “disability friendliness”.

As a result, tangible changes have been made. Several access ramps, handrails and doors were added in the psychiatric hospital wing to allow easier access to wheelchairs to the buildings, and eight new dedicated parking spots have been created in the middle of the city hospital for large vehicles. Also, the revolving doors of the main entrance to the main hospital have been replaced: they were not easily accessible to electric wheel chairs.

### Summary

Table 1

**Table 1:** Summary of the barriers, interventions and reasonable adjustments of the project

Barriers & Working group	Before the interventions	Interventions & Reasonable adjustments	After the interventions
1. Reduce quality of care and poor healthcare coordination Patient care path	Long waiting time in the ES Inadequate care Sent home without proper care No information on medical care No coordination of the patient care path Transfer home or in supported residential accommodation without coordination or warning, sometimes during the night.	A simplified emergency admission procedure A disability admission sheet A central phone number for ES admissions Shorter waiting time in ES 60% ID-physician position 100% ID nurse case manager Close collaboration with the “Pain Network” Creation of the ID outpatient clinic once a week	The disability admission sheet is used throughout the HUG and by families and every supported residential accommodation in Geneva. It is available on the internet. The central phone number has been given to families and supported residential accommodations and is used very adequately by all partners. The ID-physician position is made permanent The nurse case manager plays a key role in facilitating the communication, counselling and organising the hospital stay. No transfer of patient without warning or during the night An average of four specific consultations for PWID each week take place.
2.Poor communication and information transmission Communication	ID-Patient and families or carers not listened to by HCP No information on medical care Transfer home or in supported residential accommodation without coordination or warning, sometimes during the night.	Dedicated Internet webpage Video on the project Easy to read and understand accessible information on medical procedures	A phone call to families and carers before transfer No transfer of patient without warning or during the night. Webpage is user’s friendly and continuously improved and up-to dated The ID admission sheet is used throughout the Hospital and in all the supported residential accommodations as well as to families
3.Lack of training of healthcare professionals Human resources	HCP not aware of the specific needs of ID patient They do not know how to care for nonverbal patient with challenging behaviour.	A flash training of fifteen minutes for doctors, chief and senior nurses A two-hour session for emergency-HCP and professionals in sensitive departments A five-day training program for HCP and hospital staff Specific training program for student nurses	Training of more than 150 HCP in 2017 Participation and professionalization of PWID as co-trainer Training is greatly appreciated by participants Training of HCP remains a challenge due to lack of time and needs to be developed.
4.Inaccessibility Accessibility	Some buildings of the hospital are not accessible to wheel chairs Lack of rails and revolving doors not accessible to large electric wheelchairs. Insufficient parking spaces for large vehicles for PWID	Assessment on the accessibility to all buildings Eight new dedicated parking spots Replacement of the revolving doors of the main entrance of the hospital Several access ramps, handrails and doors added	Some revolving doors from the new hospital building need to be changed as well. The signalisation of the outpatient clinic is still unsatisfactory and needs to be improved New parking spaces should be provided for the outpatient clinic in 2019

.

## Discussion

Providing equity of care for patients with intellectual and developmental disabilities in a University hospital is the challenge that has been addressed through the Disability Project. It is interesting to compare the reasonable adjustments that were made in the project to the recommendations of the literature.

Regarding the awareness barrier, the four French reports selected in the literature review [18, 19, 30, 31] recommend that awareness on health issues for PWID should be raised to suppress barriers to healthcare. Awareness is being raised on the specific needs and healthcare for PWID among newly trained nurses. The Faculty of Medicine of the University of Geneva has agreed to include a few hours’ course on communication and ID, yet it is not sufficient regarding the complexity of competencies and skills required for this population and needs to be further developed.

Through the creation of the “Disability Webpage” [29] and the video on the project with an interview of the medical chief of the hospital, the PWID’s status changed from an invisible population to a “vulnerable population with special needs that must be addressed”. PWID are now included in the HUG’s charter and the project has become part of the official “Strategic 2020 Plan”. Moreover, a multidisciplinary meeting is organised once a year in the HUG to keep all partners and families updated and to share experiences. Some obstacles have been encountered during the implementation of this project: firstly, there were objections that the project team was establishing a positive discrimination and that specialized resources for PWID would cost extra money. Secondly, the apparent lack of a critical mass of the targeted patient group appeared also as problematic because it was thought that the project referred only to a small minority of people.

Regarding the communication barrier, the specific admission sheet has been filled in by all supported residential accommodations and families, for almost every PWID in Geneva. This is a great success, considering the variety of residential accommodations. It is now systematically used in the hospital and has significantly improved the communication between the partners. Yet it is estimated that an average of 10 % of PWID are not identified and that they are unknown to the network of associations and supported residential accommodations: These patients need to be made aware of their rights and helped to access a good quality of care. This will be one of the next aims of the ID physician and ID nurse once the Disability Project becomes a program of the University Hospital in 2019.

Regarding the reduced quality of care and poor healthcare coordination barrier, another challenge was the difficulty for professionals from supported residential accommodations and from social or medical backgrounds to communicate, as they do not share the same culture regarding PWID. Many families and social care providers still remember the time when challenging behaviours systematically led the patient to a closed psychiatric ward. The leadership team of the Disability Project has worked hard to re-establish trust between partners and to make all professionals and families work together. Another important issue is that PWID are still stigmatized as “psychiatric patients” and therefore have little or more difficult access to somatic services. They are still too often considered as having challenging behaviours related to their disability and therefore reoriented into the psychiatric emergency ward, where they do not get proper somatic investigations through lack of awareness from the hospital staff.

Regarding the lack of training of healthcare professionals barrier, the four French reports selected in the literature review [18, 19, 30, 31] and the ASSM reports [14, 32] also recommend that specialized referral centres should be developed; that specialised physicians and nurses should be identified in acute care settings as referral and support; that coordinated care path should be organised. The project has obtained the creation of two positions, for a physician and a nurse. This has contributed to provide good care for PWID for approximately 100 patients over the year 2016, over 400 in 2017 and 517 in 2018. The ID physician position and the ID nurse case manager are unique in Switzerland, and their role must be validated by research and statistics, to demonstrate their added value. The collaboration with all the partners involved in PWID’s health care emphasized that social workers and care workers should also be trained on health issues, particularly on pain detection and assessment. This has led to a partnership between the University of Applied Sciences and the association of supported residential accommodations in Geneva. A training program is being developed to address this issue. In the future, this program will also include family care givers and PWID.

The literature [13, 17, 18, 28, 29] recommends that epidemiologic studies should be promoted on the specific health needs, and that PWID should be included in prevention and health promotion programs. One of the main pitfalls encountered by the project leader was the lack of data regarding PWID in Switzerland. “Intellectual and developmental Disability” is not clearly defined in practice, particularly in French where the word “Handicap mental” has no precise definition either in the “Classification Statistique Internationale des Maladies et des Problèmes de Santé Connexes” (CIM-10) [33] or the “Diagnostic and Statistical Manual of Mental Disorders” (DSM-5) [34]. That is partly why the identification of PWID by screening is difficult. To access relevant data, a research team has been created in 2017. It is collecting data on epidemiology and prevalence of hospitalisation of PWID. However, the identification of PWID is still critical and needs to be improved: an efficient flagging system is one of the main challenges that will have to be met during the next phase of the Disability Project. Another perspective is that there is still a lot of work to do to develop the prevention and health promotion programs for PWID, as they are too often not included in the existing programs that are not adapted to them.

Regarding the patient care path organisation, each of the reasonable adjustments made this easy and quick in the ES. HCP are trained to take care of PWID and they can obtain support from the ID nurse or ID physician. Yet, it is still very difficult to get HCP to take any time off hospital work to participate in the specific ID training. So, the focus is made on shortened training sessions of one or two hours in the units during the handovers. The ID nurse and two or three co-trainers with ID visit the sensitive units to raise awareness and give some important information and take-home messages. The sensitive units are identified through the complaints of families, patients, carer or HCP. The ID nurse is well identified and is called daily by the ES staff and other sensitive units to advise professionals on how to communicate with the patients and what are the specific needs, as well as to help families, patients and carers to obtain the information and care that they need. She works on adapting various medical and care procedures for the PWID, she coordinates the interventions, specific examinations and surgeries. Among her clinical work with ID patient, the ID physician collects data and conducts researches on ID’s potential health issues in the future. They both conduct a thorough assessment of the project and collect satisfaction questionnaires systematically from PWID or from families and carers when the PWID cannot complete it.

Thanks to the good care of PWID in the hospital and the satisfaction of all partners, the Disability Project became in June 2016 a part of the policy plan of the hospital. It has now a strong institutional support from the management. The position of the ID-physician and of the ID-nurse have been made permanent and a closer collaboration has been established between the different partners. In addition to the reasonable adjustments that have already taken place, the Disability Project has initiated an out-patient clinic specialized for PWID. The development of this specific out-patient clinic takes advantage of all the different expertise and technical board of the hospital. It has developed an ambulatory consultation inside the HUG with a nurse trained in using nitrous oxide and other induced-pain prevention means. The ID nurse accompanies the patient in each specialised consultation within the hospital to facilitate the care of PWID.

These measures should reduce hospital admissions, unnecessary emergency admission and duration of hospitalisation, and this will be evaluated through the analysis of the satisfaction questionnaires, in a further phase. Thus, it is expected that the cost of the ID physician and nurse position will be counterbalanced. Finally, the current political movement in favour of a better health for PWID, particularly regarding the ageing of this population, has led to a new project to create a Health and Disability network outside the hospital in Geneva.

## Conclusion

The leadership team launched the Disability Project in 2012 without really knowing whether the desire to provide good care for PWID was just wishful thinking of a small group of HCPS or a real need for PWID, families and other partners. From the very first multidisciplinary meeting, PWID, families, carers, associations and professionals immediately showed a high level of interest. Families and associations enthusiastically embarked on the breach opened by the project team in 2012 and worked hard to support the project until it became part of the hospital’s strategic program.

Thanks to the constant support of the hospital’s medical director and each partner, the Disability Project has reached a level of success greater than what the leadership team originally expected. It has shown that reasonable adjustments can be made in an acute care setting to provide good care for PWID and their families.

### Achievements

The most important achievement of the Disability Project is the creation of the two positions of the ID Nurse Case Manager and the ID Physician, which have become essential for the smooth running of the project. These two highly skilled professionals play a critical role, training HCP about PWID’s health issues, advising and supporting hospital staff, and coordinating the interventions during hospitalisations. The second main achievements are the short waiting time, the central phone number and the disability admission sheet implemented in the Emergency Service and adopted by all the partners involved.

Theses reasonable adjustments indicate a crucial change in the global attitude of the hospital staff towards PWID and show the dedication of the management of the hospital to this cultural evolution.

### Unexpected benefits

The project has created tremendous new networking opportunities. One of the greatest benefits, though unplanned initially, has been to enable a better communication between the partners and to create a network of motivated people ready to volunteer for the wellbeing of PWID. Indeed, a strong partnership between HCP, supported residential accommodation providers, politicians, families and PWID is the key to the success of such a project.

### Evaluation

The evaluation of the Disability Project started in 2016 and is still ongoing. Because the goal of the project is to provide good care for PWID in an acute care setting, patient satisfaction has been identified as the most relevant element to assess, the key indicator of the quality of care: A satisfaction’s questionnaire assesses the efficiency of the actions and the global experience of the admission and the stay at the hospital. These questionnaires are filled in on a voluntary basis by the PWD themselves, when possible, or more often by the person accompanying the PWID, family carers or residential accommodations professionals.

The training program for HCP is also currently still undergoing evaluation and the feedback has been so far very positive.

### Relevance

The relevance of the Disability Project has been evaluated by the managers of the strategic planning of the hospital. It has been identified as one of the fifteen key projects of the Quality Program 2020.

### Perspectives

An important consequence of the success of the Disability Project has been the creation of a specific disability out-patients clinic directed by the ID physician. This new consultation is opened every Monday afternoon in the hospital and is already overbooked at the time of this writing.

Furthermore, the ID physician and the leadership manager of the project, with the support of the state department of health, are working together to create a Health and Disability Network in the city with a specific consultation “Handiconsult”, on purpose of preventing hospitalisation through better and earlier detection of health problems.

To conclude this formidable adventure, it is the hope and dearest wish of the leadership team that similar projects should be implemented in other hospitals or health facilities around the world. In such an event, it will be necessary to consider that the procedures and organisation of such a project should be adapted to the local culture and socio-economic environment. It is our belief that providing better care for PWID can be achieved anywhere with a handful of committed people and enough political support. In the long run, improving the health care of people with disabilities, raising the awareness on their specific health needs, improving the training of health care professionals to such complex situations will eventually benefit all patients whether they are disabled or not.

## Abbreviations

ASSM: Académie Suisse des Sciences Médicales
CIM-10: Classification Statistique Internationale des Maladies et des Problèmes de Santé Connexes
DSM-5: Diagnostic and Statistical Manual of Mental Disorders
ES: Emergency Service
HCP: Health Care Professionals
HUG: Hôpitaux Universitaires de Genève
ID: Intellectual Disability
PWID: Patients With Intellectual and developmental Disabilities
UNAPEI: Union nationale des associations de parents, de personnes handicapées mentales et de leurs amis
WHO: World Health Organisation

## References

[1] WHO. World report on disability. Geneva: World Health Organization; 2011.

[2] WHO. Consulté le In: 8 18, 2018, Sur infographics [internet]. 2013. Available from: http://www.who.int/disabilities/infographic/en/.

[3] Death by indifference: following up the treat me right! London: Mencap; 2007.

[4] Death by indifference: 74 deaths and counting – a Progress report 5 years on. London: Mencap; 2012.

[5] Michael J, Richardson A (2008). Healthcare for all: the independent inquiry into access to healthcare for people with learning disabilities. Tizard Learning Disability Review.

[6] Wieland A (2016). Ungewohnte Patienten und Patientinnen-Menschen mit einer geistigen Behinderung im Spital [Internet]. Fribourg.

[7] Statistique des institutions médico-sociales. La situation des personnes handicapées en institution. Département fédéral de l’intérieur, DFI [Internet]. Office fédéral de la statistique; 2012. Available from: https://www.bfs.admin.ch/bfs/fr/home/statistiques/sante/systeme-sante/institutions-specialisees.assetdetail.348707.html. Accessed 7 Mar 2019.

[8] The World Health Report 2001: Mental health: new understanding, new hope. World Health Organization; 2001 p. 2.

[9] Maulik PK, Mascarenhas MN, Mathers CD, Dua T, Saxena S (2011). Prevalence of intellectual disability: a meta-analysis of population-based studies. Res Dev Disabil.

[10] Azéma B, Martinez N (2005). Les personnes handicapées vieillissantes: espérances de vie et de santé; qualité de vie. Revue française des affaires sociales.

[11] Morin D, Mérineau-Côté J, Ouellette-Kuntz H, Tassé MJ, Kerr M (2012). A comparison of the prevalence of chronic disease among people with and without intellectual disability. American journal on intellectual and developmental disabilities.

[12] Coûts et financement du système de santé 2015: données définitives. Département fédéral de l’intérieur, DFI [Internet]. OFS; 2017. Available from: https://www.bfs.admin.ch/bfs/fr/home/statistiques/sante/cout-financement.html. Accessed 7 Mar 2019.

[13] Convention on the Rights of Persons with Disabilities (CRPD)|United Nations Enable [Internet]. [cited 2019 Jan 20]. Available from: https://www.un.org/development/desa/disabilities/convention-on-the-rights-of-persons-with-disabilities.html#Fulltext. Accessed 7 Mar 2019.

[14] Traitement médical et prise en charge des personnes en situation de handicap: Directives médico-éthique de l’ASSM. Basel: Académie Suisse des Sciences Médicales; 2008.

[15] Learning from the past, setting out the future: developing learning disability nursing in the United Kingdom. London: Royal College of Nursing; 2011.

[16] Sowney M, Barr OG (2006). Caring for adults with intellectual disabilities: perceived challenges for nurses in accident and emergency units. J Adv Nurs.

[17] Sowney M, Barr OG (2007). The challenge for nurses communicating with and gaining valid consent from adults with intellectual disabilities within the accident and emergency care service. J Clin Nurs.

[18] Pour une santé accessible aux personnes handicapées mentales. Paris: Union nationale des associations de parents, de personnes handicapées mentales et de leurs amis; 2013.

[19] Haute Autorité de Santé - Audition publique “Accès aux soins des personnes en situation de handicap” du 22 au 23 octobre 2008 [Internet]. [cited 2018 Jan 10]. Available from: https://www.has-sante.fr/portail/jcms/c_736311/fr/acces-aux-soins-des-personnes-en-situation-de-handicap-rapport-de-la-commission-d-audition-publique.

[20] Bowness B (2014). Improving general hospital care of patients who have a learning disability.

[21] Northway R, Jenkins R, Holland-Hart D (2017). Training of residential social care staff to meet the needs of older people with intellectual disabilities who develop age-related health problems: an exploratory study. J Appl Res Intellect Disabil.

[22] Northway R, Holland-Hart D, Jenkins R. Meeting the health needs of older people with intellectual disabilities: exploring the experiences of residential social care staff. Health & social care in the community. 2017:923–31. 10.1111/hsc.12380 27580975

[23] Northway R, Rees S, Davies M, Williams S (2017). Hospital passports, patient safety and person-centred care: a review of documents currently used for people with intellectual disabilities in the UK. J Clin Nurs.

[24] Evenhuis HM, Gorter E, Von d, Möhlen-Tonino M, Meijer MM, Den Ouden WJ, Scholte FA (2003). Manifeste Européen. Niveau élémentaire de soins de santé pour les personnes présentant un handicap intellectuel.

[25] Balogh R, Ouellette-Kuntz H, Bourne L, Lunsky Y, Colantonio A (2008). Organising health care services for persons with an intellectual disability (2008). Cochrane Database Syst Rev.

[26] Factsheets [Internet]. World Health Organization; 2013 p. 352. Available from: http://www.who.int/mediacentre/factsheets/fs352/en/. Accessed 7 Mar 2019.

[27] Discrimination [Internet]. London: RCN; 2013. Available from: https://www.rcn.org.uk/get-help/rcn-advice/discrimination. Accessed 7 Mar 2019.

[28] Hogg J, Lucchino R, Wang K, Janicki M (2001). Healthy ageing - adults with intellectual disabilities: aging and social policy. J Appl Res Intellect Disabil.

[29] Accueillir un patient en situation de handicap [Internet]. [cited 2018 Oct 21]. Available from: https://www.hug-ge.ch/accueillir-patient-situation-handicap

[30] Hôpital Handicap : Pour une amélioration continue de la qualité dans l’accueil et les soins pour les personnes handicapées à l’hôpital: Manuel d’accréditation de l’ANAES. ANAES; 2002.

[31] Rapport triennal de l’Observatoire national sur la formation, la recherche et l’innovation sur le handicap. Paris: Observatoire national sur la formation, la recherche et l’innovation sur le handicap; 2011.

[32] Droit des patientes et patients à l’autodétermination: Principes médico-éthique de l’ASSM. Basel: ASSM; 2005.

[33] CIM-10 Version:2008 [Internet]. [cited 2018 Oct 21]. Available from: http://apps.who.int/classifications/icd10/browse/2008/fr

[34] DSM-5 [Internet]. [cited 2018 Oct 21]. Available from: https://www.psychiatry.org/psychiatrists/practice/dsm

